# Characterization of Focal Liver Lesions Indistinctive on B Mode Ultrasound: Benefits of Contrast-Enhanced Ultrasound

**DOI:** 10.1155/2017/8970156

**Published:** 2017-04-11

**Authors:** Yi Dong, Feng Mao, Jiaying Cao, Peili Fan, Wen-Ping Wang

**Affiliations:** ^1^Department of Ultrasound, Zhongshan Hospital, Fudan University, Shanghai 200032, China; ^2^Shanghai Institute of Imaging, 180 Fenglin Road, Shanghai 200032, China

## Abstract

*Aim.* The aim of this prospective study was to evaluate the additional value of contrast-enhanced ultrasound (CEUS) in identifying and characterizing of focal liver lesions (FLLs) that are indistinctive on B mode ultrasound (BMUS).* Methods.* The study focused on 70 consecutive patients (male 46, female 24; mean age, 53.1 years ± 10). All lesions were detected by MRI but could not be clearly visualized by BMUS. CEUS was performed by injected SonoVue® (Bracco Imaging Spa, Milan, Italy) as a quick bolus into the antecubital vein. All lesions were proved by pathologic and MRI findings as primary or metastatic hepatic malignancies.* Results.* On CEUS, 45 (64.2%) FLLs displayed arterial hyperenhancement and 55 (78.5%) lesions showed hypoenhancement in portal venous and late phase (PVLP). Homogeneous and complete hyperenhancement pattern during the arterial phase is highly suspicious for HCC in liver cirrhosis (96.8%). Arterial isoenhancement and early washout during PVLP are characteristic for metastasis (73.3%). For recurrence lesions, arterial hyperenhancement and isoenhancement during PVLP are more common (60%).* Conclusion.* CEUS may provide added diagnostic values in FLLs appearing indistinctive on BMUS. Presence of early arterial enhancement and washout during PVLP may be helpful for detection of those lesions.

## 1. Introduction

Incidentally detected focal liver lesions (FLLs) are more commonly encountered in daily abdominal imaging practice, which may need further clinical investigations [1]. Once a FLL is detected, it is crucial to characterize it with the aim to confirm or rule out HCC or other malignancies [2].

B mode ultrasound (BMUS) is the most popular used diagnostic tool available for the assessment of FLLs. Therefore, a common practice is to use targeted BMUS immediately to assess the indeterminate small lesion found with CT or MRI. Otherwise, further percutaneous biopsy or radiofrequency ablation (RFA) will always be guided by real-time BMUS. Short-term BMUS follow-up may be needed to further characterize the lesion [3]. Previous study showed the detection and characterization ability of BMUS is higher if the lesion detected with CT is >5 mm [3]. However, BMUS still shows low specificity and low accuracy for optimal characterization of small FLLs [4, 5]. Detection and differentiation of benign or malignant FLLs on a significant cirrhotic background can be a challenge [6, 7]. Detection of isoechoic or small (less than 1 cm in diameter) FLLs is also difficult with BMUS [8]. As reported in the literature, some metastases are still undetected in BMUS, which is currently the benchmark staging method in patients with colon cancer [9].

In recent years, contrast-enhanced ultrasound (CEUS) has demonstrated a significant efficacy and dramatic improvement in either detection or characterization of FLLs. Previously reported data for the differentiation of FLLs showed good accuracies for CEUS, ranging from 85% to 91%, with moderate interobserver agreement [10]. CEUS enjoyed a real-time diagnostic accuracy similar to that of computed tomography (CT) and magnetic resonance (MR) imaging, without the use of ionizing radiation or nephrotoxic agents [11–14]. CEUS was also proved to be possible to achieve a significant improvement in sensitivity for metastasis detection or excluding metastases in cancer patients with MDCT evidence of subcentimetric, indeterminate focal liver lesions [3], which is also comparable to that of CT or MRI [15–17].

The European Federation of Societies for Ultrasound in Medicine and Biology (EFSUMB) guidelines and comments of the guidelines recommend the use of CEUS to diagnose suspected lesions identified in a background of chronic hepatitis or liver cirrhosis, also in patients with a known history of malignancy [18, 19]. CEUS is discussed for the recall and characterization of FLLs ≥ 1 cm based on contrast-enhanced imaging techniques with the use of vascular contrast media [2]. However, only a few studies have been conducted using CEUS to determine the enhancement pattern of those atypical or indistinctive lesions on BMUS.

The purpose of our current prospective study was to assess the additional value of the CEUS in identification and characterization of those histologically confirmed FLLs which were indistinctive or undetermined on BMUS.

## 2. Materials and Methods

### 2.1. Institutional Board Approval

This prospective study was approved by our institutional review board. All patients gave their full informed consent before the CEUS examination. The procedure followed was in accordance with the Declaration of Helsinki.

### 2.2. Patients

Between Feb 2012 and June 2016, 1250 patients were referred to our institution for liver CEUS assessment. Among them, 70 consecutive patients (24 women and 46 men; age range: 22–84 years, mean: 55 years ± 13) were detected by MRI but could not be clearly visualized by BMUS (Table 1). The patients' inclusions criteria were as follows: all lesions were detected as malignant FLLs by MRI in the last month; patients need further ultrasound guided biopsy or minimal invasive treatment (such as RFA); lesions were nonvisible on BMUS ultrasound during the regular clinical procedure. The exclusion criteria were age < 18 year, recent cardiac infarct, and known allergic reactions to CEUS contrast agents.

The final diagnoses for 43 patients were based on histopathologic results obtained from ultrasound guided 18-gauge core-needle biopsy (*n* = 6) or surgery (*n* = 37). For the remaining 27 patients without histologic confirmation, typical appearance of these lesions on contrast-enhanced MR images, with systematic follow-up (at least 12 months), was considered the reference standard.

### 2.3. Examination Technique

Two experienced radiologists (more than 15 years' experience in CEUS of the liver), who were aware of the patients' clinical histories, performed ultrasound scanning with a Siemens S2000 ultrasound system (Siemens AG, Erlangen, Germany, 4C-1 transducer) or LOGIQ E9 ultrasound system (GE Healthcare, Milwaukee, WI, USA, C1-5-D transducer).

A baseline ultrasound examination, including grey scale and color flow imaging analysis, was performed. After detailed evaluation of prior MR images, BMUS was used to examine the whole liver and searched for the suspected FLL. Intrahepatic anatomic structures such as cysts, blood vessels, gallbladder, or scars were used as references. Optimized instrument settings were used to acquire more clear visualization and to find the proper location of lesions, such as the adjustment of focal zones, field of view, dynamic range, and application of harmonic imaging. If the FLL was not visible or could not be differentiated from regenerative or cirrhosis nodules, it was regarded to be indistinctive.

A 2-step strategy with repeated injection of SonoVue was applied in all 70 patients. First targeted CEUS were performed to detect any hypoenhanced lesion during portal venous or late phase (PVLP), with SonoVue (Bracco, Milan, Italy) as contrast agent, which was injected intravenously as a 2.4 mL bolus followed by 5 mL of normal sterile saline flush via a 22-gauge peripheral intravenous cannula. Low mechanical index (MI) ranging from 0.05 to 0.08 was used for real-time CEUS imaging. Each examination lasted for at least 5 minute after bolus injection. Then a further 2.4 mL bolus of SonoVue was administered to focus on the detailed contrast enhancements of FLLs detected, with an interval time of at least 15 minutes to allow for clearance of the previous injected contrast agents.

Digital cineloops were digitally stored as raw-data in a PC-based workstation connected to the ultrasound equipment.

### 2.4. Image Analysis

Before CEUS, grey scale echogenicity of the lesions was observed in comparison with adjacent liver parenchyma. We classified those FLL as being minor hyperechoic, isoechoic, and minor hypoechoic.

Immediately after the injection of CEUS agents, 2 examiners evaluated by consensus the dynamic enhancement pattern of each lesion in comparison to surrounding liver parenchyma. The pattern of enhancement throughout the arterial, portal venous, and late phases was observed according to the EFSUMB guideline [18, 19]. The CEUS enhancement patterns during arterial phase were subjectively classified as (1) diffuse homogeneous (entire lesion enhanced rapidly and uniformly); (2) diffuse inhomogeneous (heterogeneous enhancement of the whole lesion); (3) rim-like (peripheral enhancement). Special attention was paid to presence or absence of early arterial enhancement and to the detection of any PVLP washout area of contrast agents.

Before and after CEUS, a 4-point scale was used to grade detection confidence: (1) distinctive; (2) probably visible; (3) poorly visible; (4) invisible [20].

### 2.5. Statistical Analysis

Statistical analysis was performed with a computer software package (SPSS, version 15.0, IBM corporation, Armonk, USA). The improvement in diagnostic confidence was assessed by receiver operating characteristic (ROC) curves analysis. For all tests a *P* value < 0.05 was considered to indicate a statistically significant difference.

## 3. Results

### 3.1. Final Diagnosis of FLLs

Consequently, 70 FLLs not visible on BMUS in 70 patients were included in this study (Table 2). Single malignant FLLs were detected in 52 patients and multiple lesions in 18 patients. For multiple FLLs, only the biggest one was evaluated during our current study. Final diagnosis proved 35 primary HCCs, 20 recurrent HCCs, and 15 metastasis malignancies. The median size of those FLLs was 16 mm (size range: 6–30 mm; mean ± SD: 14.6 ± 6 mm).

### 3.2. Detection Rate of FLLs

All those lesions were isoechoic (*n* = 45), slightly hyperechoic (*n* = 11), or slightly hypoechoic (*n* = 14) with indistinctive margins on BMUS. After first CEUS procedure, 64.2% of FLLs showed hyperenhancement during arterial phase and 78.5% lesions showed hypoenhancement during PVLP (Table 3).

Comparing the 4-point scale classification results between BMUS and CEUS, among 70 FLLs diagnosed on MR imaging, BMUS identified 11 (15.7%) lesions as poorly or probably visible lesions. CEUS detected an additional 59 (84.3%) lesions (Table 4), which was significantly higher than BMUS (*P* < 0.05).

### 3.3. CEUS Enhancement Pattern (Repeated Injection)

**Figure 1:**
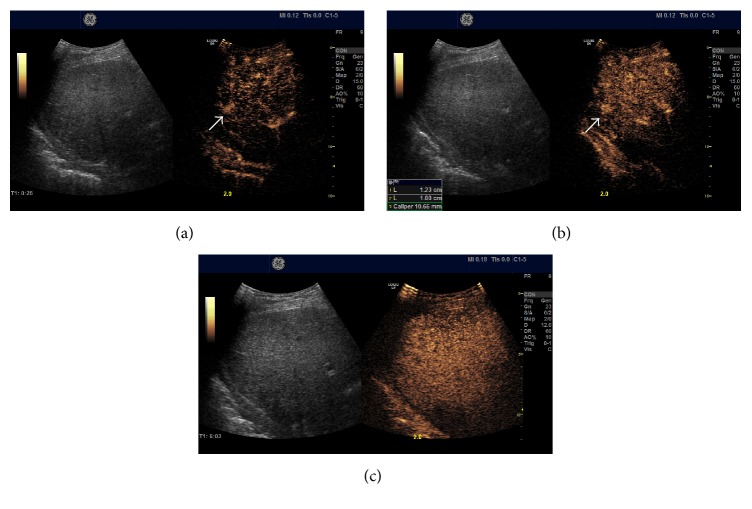
Small HCC (10 mm) nonvisible on B mode ultrasound but was hyperenhanced during arterial phase (a) and portal venous (b) phase. It was slightly hypoenhanced in the late phase (c) on contrast-enhanced ultrasound. The arrow in (a) and (b) refers to the hyperenhanced small HCC lesion.

**Figure 2:**
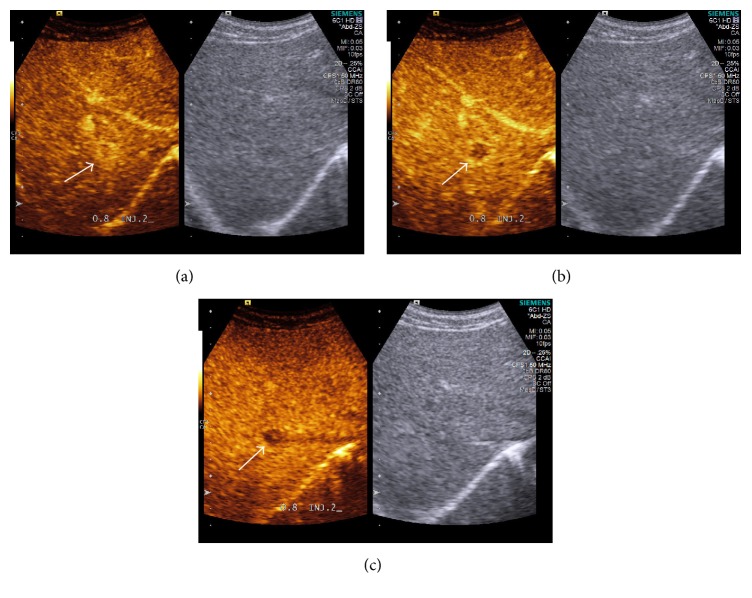
Small metastasis lesion from colon (15 mm) not clearly visible on B mode ultrasound showed rim-like hyperenhancement during arterial phase (a). Clear hypoenhancement during the portal venous (b) and late phase (c) on contrast-enhanced ultrasound. The arrow in the figure refers to rim-like hyperenhanced small metastasis lesion during arterial phase and showed clear hypoenhancement during the portal venous.

**Figure 3:**
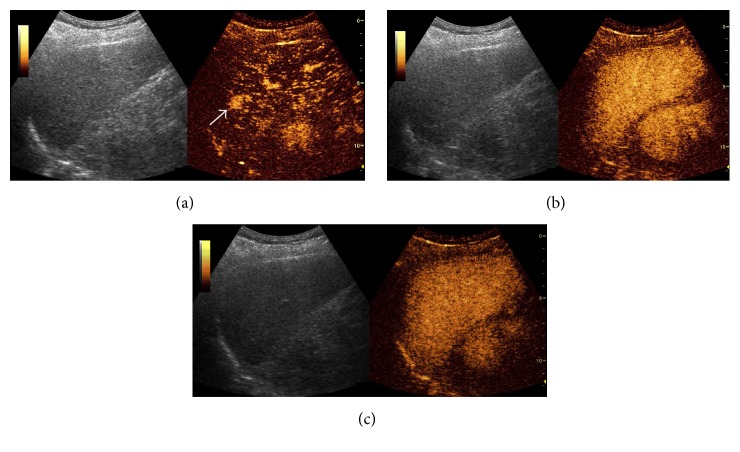
Small recurrent lesion (15 mm) indistinctive on B mode ultrasound showed homogeneous hyperenhancement during arterial phase (a). Isoenhancement during the portal venous (b) and late phase (c) on contrast-enhanced ultrasound. The arrow in (a) refers to homogeneously hyperenhanced small recurrence lesion during arterial phase.

After repeated injection of SonoVue, the contrast enhancements of FLLs were evaluated as well focussing on the lesion detected, during the arterial phase (10–30 seconds), portal venous (30–120 seconds), and late phases (120–300 seconds). On CEUS, homogeneous and complete hyperenhancement pattern during the arterial phase is highly suspicious for HCC in liver cirrhosis patients (96.8%) (Figure 1). Arterial isoenhancement early washout during PVLP is characteristic for metastasis malignancies (54.7%) (Figure 2). Arterial hyperenhancement and isoenhancement during PVLP are more common for recurrent HCCs (54.7%) (Figure 3) (Table 5).

## 4. Discussion

According to the EFSUMB guideline, CEUS allowed the characterization of most of FLLs by analysis of the arterial, portal venous, and late phases [18, 19]. Previously studies reported that CEUS has a high diagnostic accuracy for the differential diagnosis of FLLs based on description of tumor-specific enhancement patterns, obtaining a sensitivity of 90%, a specificity of 99%, and an accuracy of 89% for the diagnosis for the discrimination of malignant and benign FLLs when compared with BMUS [21]. Depending on the presence of early arterial enhancement, and by detection of PVLP washout, our results indicated that CEUS may play a confirmatory role in the detection of those indistinctive FLLs on BMUS. Detailed scanning during the late phase of CEUS enables better detection of malignant FLLs [22].

Ultrasound contrast agents are strictly intravascular, without diffusion into the interstitial space. This explains that evidence of washout during PVLP is the most important feature in the differentiation of FLLs [18, 19]. Also, it allows for discrimination between types of malignancy [14]. In our current study, early and complete washout during PVLP is typical for metastases FLLs. However, HCCs often showed a relatively slower washout and 57.1% of HCCs still are isoenhanced during portal venous phase.

Echogenicity of FLL depends on its size and on the echo difference compared with surrounding liver parenchyma. BMUS recognition of HCC in liver cirrhosis can be difficult if the echo texture is very inhomogeneous [23]. Although many HCCs demonstrate arterial phase enhancement and moderate washout during PVLP, some small HCCs are seen only during the arterial phase. During the first CEUS, the primary CEUS feature to be searched for detection of HCCs in cirrhosis is the hyperenhancement in the arterial phase.

Meanwhile the detection of subsequent hypoenhancement during portal or late phase is also requested to definitively establish the diagnosis of HCC [2, 18, 19, 24]. The rates of arterial hyperenhancement in HCCs are reported to be increasing with size: in lesions ≤ 2.0 cm and equal to 3.0 cm, they are between 40 and 70%, respectively [25, 26]. Most of FLLs are smaller than 2 cm in our current research; as a result, most of HCCs in our current study showed homogeneous and complete hyperenhancement pattern during the arterial phase in liver cirrhosis. The washout tends to start later in HCC [27, 28]. In our study, 51.3% of lesions were hypoenhanced after 180 s after injection of the contrast agent. Therefore, for those indistinctive HCCs on BMUS, it is important to prolong the observation of contrast enhancement in cirrhosis for up to 4 minutes [2].

The presence or absence of liver metastases plays a vital important role in the choice of therapy. Therefore, it is crucial to have accurate preoperative methods for the detection of liver metastases [29]. Despite advances in modern imaging techniques, assessing the presence of liver metastases remains challenging; currently there is no reliable method for detecting small, occult liver metastases [30, 31]. In our study, liver metastases are found on CEUS in 21.4% of patients which are indistinctive on BMUS. For those metastasis malignant lesions, isoenhancement during arterial phase and early washout during PVLP are more common. Metastases consistently show rapid washout (<60 seconds) [32]. CEUS provides a fast and reliable diagnosis, making any other further imaging investigations unnecessary. The mean diameter of those metastasis lesions is 12 mm; therefore these patients are being early diagnosed and referred for further treatment. According to the up-to-date standard, CEUS should be recommended in the follow-up of patients with colon cancer in addition to BMUS [33].

For recurrent HCC lesions, arterial hyperenhancement and isoenhancement during PVLP are more common. A possible explanation for nonvisualization of recurrent lesions on BMUS images in our study may be the interference of scars from previous operation or minimal invasive treatment (such as radiofrequency ablation and percutaneous ethanol injection) or that neoangiogenic process may not be fully developed in smaller recurrent lesions [34, 35].

## 5. Conclusion

In conclusion, 2-step CEUS may provide added diagnostic values in those FLLs appearing indistinctive on BMUS. Presence of early arterial enhancement and washout area during PVLP may be helpful for increasing the detection rate of those lesions.

## Figures and Tables

**Table 1:** Baseline characteristics of patients.

Characteristic	Patients
	(*n* = 70)
Age (year)	
Mean ± SD	55 ± 13
Range	22–84
Male/female	46/24
Underlying liver diseases	
Cirrhosis (viral/alcohol)	27 (25/2)
Fibrosis (viral/alcohol)	26 (25/1)
Previous tumor history	15
None	2
ΑFP (ng/mL)	
≤20, *n* (%)	46 (65.7%)
21–200, *n* (%)	17 (24.3%)
>200, *n* (%)	7 (10%)
CA 19-9 (*μ*/mL)	
≤4.9, *n* (%)	6 (8.5%)
>4.9, *n* (%)	64 (91.5%)
Final diagnosis	
Liver surgery	37
Core needle biopsy	6
MR images follow-up	27

AFP: alpha-fetoprotein; CA19-9: carbohydrate antigen 19-9; HCC: hepatocellular carcinoma; *n*: number.

**Table 2:** Baseline characteristics of FLL included.

Characteristic	FLL lesions
	(*n* = 70)
Diameter (mm)	
Mean ± SD	14.6 ± 6
Range	6–30
≤20 mm (%)	63 (90%)
20–30 mm (%)	7 (10%)
Location	
Left lobe, *n* (%)	18 (25.7%)
Right lobe, *n* (%)	41 (58.5%)
Left & right lobe, *n* (%)	11 (15.8%)
Lesion number, *n* (%)	
*n* = 1	52 (74.3%)
*n* = 2	12 (17.1%)
*n* > 3	6 (8.6%)
Final diagnosis, *n* (%)	
Primary HCC	35 (50%)
Recurrent HCC	20 (28.5%)
Metastasis	15 (21.4%)

FLL: focal liver lesion.

**Table 3:** Detection of FLL not visible on conventional BMUS.

Characteristic	FLL lesions
	(*n* = 70)
BMUS, *n* (%)	
Isoechoic	45 (45.4%)
Slightly hyperechoic	11 (46.8%)
Slightly hypoechoic	14 (7.8%)
Detected by first CEUS	
Arterial hyperenhancement, *n* (%)	45 (64.2%)
PVLP hypoenhancement, *n* (%)	55 (78.5%)

FLL: focal liver lesion; BMUS: B mode ultrasound; CEUS: contrast enhanced ultrasound.

**Table 4:** Results of 4-point scale grade of BMUS and CEUS in 70 indistinctive FLLs.

Four-point scale of indistinctive lesions	BMUS	CEUS
Grade 1, invisible	8	0
Grade 2, poorly visible	3	5
Grade 3, probably visible	0	15
Grade 4, distinctive	0	50

FLL: focal liver lesion; BMUS: B mode ultrasound; CEUS: contrast enhanced ultrasound.

*P* = 0.008 (Fisher's exact test).

**Table 5:** Contrast enhancement features of 70 indistinctive lesions BMUS.

Characteristic	Primary HCCs	Recurrence HCCs	Metastasis
	(*n* = 35)	(*n* = 20)	(*n* = 15)
Arterial phase enhancement, *n* (%)			
Hyperenhanced	26 (74.2%)	16 (80%)	3 (20%)
Isoenhanced	9 (25.8%)	4 (20%)	12 (80%)
Type of arterial phase enhancement, *n* (%)			
Diffuse homogeneous enhancement	25 (71.4%)	12 (60%)	1 (6.7%)
Diffuse inhomogeneous enhancement	10 (28.6%)	7 (35%)	2 (13.3%)
Rim-like hyperenhancement	0	1 (5%)	12 (80%)
Portal venous phase enhancement, *n* (%)			
Isoenhanced	20 (57.1%)	15 (75%)	4 (26.7%)
Hypoenhanced	15 (42.9%)	5 (25%)	11 (73.3%)
Late phase enhancement, *n* (%)			
Isoenhanced	2 (5.7%)	12 (60%)	1 (6.7%)
Hypoenhanced	33 (94.2%)	8 (40%)	14 (93.3%)

BMUS: B mode ultrasound; HCC, hepatocellular carcinoma; CEUS: contrast enhanced ultrasound.
